# Long Noncoding RNA LOC441178 Reduces the Invasion and Migration of Squamous Carcinoma Cells by Targeting ROCK1

**DOI:** 10.1155/2018/4357647

**Published:** 2018-10-01

**Authors:** Kai Xu, Hurong Tian, Shouyong Zhao, Daoying Yuan, Licheng Jiang, Xianbin Liu, Bo Zou, Jin Zhang

**Affiliations:** ^1^Department of Oral and Maxillofacial Surgery, Liaocheng People's Hospital, Liaocheng, Shandong 252000, China; ^2^Department of Clinical Laboratory, Liaocheng People's Hospital, Liaocheng, Shandong 252000, China

## Abstract

In recent years, long noncoding RNAs (lncRNAs) have been reported to have significant regulating effect in human cancer development. Previous studies suggested that dysregulation of lncRNA 441178 (LOC441178) is possibly associated with oral squamous cell carcinoma (OSCC). The postoperative survival time was significantly prolonged in the high-grade OSCC patients with high LOC441178 expression compared with those with low LOC441178 expression, which indicated that LOC441178 may act as a prognostic marker and as a potential tumor suppressor for OSCC. However, the biological molecular mechanisms behind these phenomena remain almost unknown. Here, our studies revealed that LOC441178 suppressed the invasion and migration of squamous carcinoma cells (SCCs). Furthermore, we found that rho-associated, coiled-coil-containing protein kinase 1 (ROCK1) is one of the functionally relevant targets of LOC441178 in squamous cells, which is negatively correlated with LOC441178 in tumor tissues from OSCC patients. In conclusion, our findings demonstrated the inhibition effect of LOC441178 on tumor in OSCC and might have potential implications for OSCC gene therapy. In conclusion, these results suggest that LOC441178 could represent a prognostic indicator for OSCC and be a new target for the diagnosis and treatment of OSCC.

## 1. Introduction

OSCC is the sixth most common malignant tumor worldwide, with 610,000 new cases and 334,000 cancer deaths in 2016 [1]. OSCC accounts for more than 90% of all mouth malignancies and approximately 40% of head and neck tumors [2]. The biological properties of OSCC include uncontrollable invasiveness, high mortality, and widespread hypoxia, which are enormous challenges to the treatment of this disease [3].

Although several mechanisms have been studied to explicate the mechanisms of migration and invasion of tumor cells, the study of the relationship between lncRNAs and cancer has only begun in the last ten years. There is already evidence suggesting that lncRNAs may play important roles in the pathogenesis and development of tumor, including OSCC [4]. In 2011, Gibb et al. presented the first lncRNA expression map for the human oral mucosa and found that 60% of the detected lncRNAs showed abnormal expression in oral precancerous lesions [5]. Afterwards, many studies investigated the function of lncRNAs in tumor progression through comparing their expressions between cancerous tissues and normal tissues. Several abnormally expressed lncRNAs have been reported by some studies in oral cancers, including UCA1 [6], HOX transcript antisense RNA (HOTAIR) [7, 8], and MALAT1 [9]. For example, HOTAIR, the first lncRNA which was found to have regulatory functions of reverse transcription, was related to OSCC tumorigenesis. The upregulation of HOTAIR in OSCC tissues was associated with tumor cell invasion and poor prognosis of OSCC patients [10].

LOC441178, a lncRNA with 2557 nucleotides, has been reported by several groups in oral carcinoma [11]. This lncRNA has been found abnormally expressed in several types of cancer, such as cervical cancer and gastric cancer, and its increase is relevant to development and metastasis of several cancers, such as colorectal and gastric cancers. A study using microarray technology found that LOC441178 was significantly downregulated in OSCC compared to normal oral mucosa tissues, suggesting that it could potentially act as a predictor of cancer diagnosis or prognosis [12]. However, the biological molecular mechanisms of these properties remain almost unknown.

In this study, we demonstrate that LOC441178 is a tumor suppressor and a potential prognostic marker of OSCC. First, we analyzed datasets from GEO database and demonstrated that the expression of LOC441178 was much lower in OSCC patients than control patients. Further investigation using cell lines revealed that LOC441178 reduced the invasive and migration abilities of SCCs through targeting ROCK1. Immunohistochemistry staining of OSCC and control tissue samples showed that the expression of ROCK1 is negatively correlated with the level of LOC441178. These findings of LOC441178 suggest that it functions as tumor suppressor and prognostic biomarker in OSCC. This study shows the significance of lncRNAs LOC441178 in OSCC development and provides clues to its molecular mechanisms.

## 2. Materials and Methods

### 2.1. Data and Date Source

All LOC441178 expression datasets were downloaded from GEO. Data were retrieved using the keywords “LOC441178” and “squamous cell carcinoma”. Two datasets were included in this study, GSE30784 and GSE9844. There are 212 samples in the first dataset; 167 of them are OSCC tissues and others are oral mucosa from healthy controls. The second dataset has 26 tongue squamous cell carcinoma tissues and 12 adjacent normal tissues.

### 2.2. Tissue Samples Collection

52 patients that were diagnosed with OSCC were included in this study. All pathological diagnoses were performed by the pathology department in the Department of Oral and Maxillofacial Surgery, Liaocheng People's Hospital. Normal oral mucosal tissues were obtained from healthy patients or adjacent tissues. All experiments were approved by the Ethics Committee at Liaocheng People's Hospital and all patients in this study have signed the informed consents.

### 2.3. Cell Lines and Cell Culture

Human squamous carcinoma lines SCC-25 were purchased from the BEIJING ZHONGYUAN LTD. All cells were cultured in DMEM medium with 10% fetal bovine serum (FBS). Cells were cultured at 37°C with 5% CO2 in an incubator. All the regents were purchased from Gibco (Grand Island, USA). The selective ROCK inhibitor, Y-27632, was purchased from Sigma-Aldrich (USA). Y27632 is a nonisoform selective ROCK inhibitor that targets the ATP-binding pocket of the ROCKs due to the high degree of homology in the kinase domain of ROCK1 and ROCK2 [13].

### 2.4. Cell Transfection

Lipofectamine 2000 (Invitrogen, USA) was used to perform cell transfections. 10 *μ*L Lipofectamine 2000 was diluted with 250 *μ*L serum free DMEM medium and incubated for 5 min at room temperature. Plasmid pcDNA3.1-LOC441178 and LOC441178 inhibitor (sequence: 5′-CAGUGAGGUAGUCUCACAA-3′) were synthesized by RiboBio Co. (Guangzhou, China). 4 *μ*g plasmid was diluted with 250 *μ*L serum-free DMEM medium. Then the Lipofectamine 2000 and plasmid were mixed and kept at room temperature for 20 min. The old culture medium was removed when the cells reached 80% confluence and 2 mL DMEM medium was added. Finally, the Lipofectamine/plasmid mixture was added to the medium. 48 hours after transfection, the OSCC cells were harvested for further experiments. Cells without transfection were used as controls. The transfection efficiency was estimated using RT-qPCR.

### 2.5. Cell Viability Assay

Cell viability was analyzed using a Cell Counting Kit-8 (Beyotime, China) 48 h after transfection. Transfected or control SCC-25 cells were inoculated in 96-well culture plates at a concentration of 3000 cells/well (100*μ*L/well). Then, each well was added with 10 *μ*L CCK-8 solution and incubated for 1 h at 37°C. Optical density at 450 nm was measured using a microplate reader.

### 2.6. Migration Analysis

The migration ability was determined by wound-healing assay using previously published methods in 6-well culture plates [14]. Transfected or untransfected SCC-25 cells were seeded in a 6-well plate at a density of 5 × 10^5^/mL. After culturing for 12 h, the cell monolayer was scratched with a pipette tip and the medium was changed. Cells were stained with crystal violet and images were captured using a microscope. After another 6 hours, other cells were stained and images were captured.

Effects of Y27632 on differently transfected SCC-25 cells were examined by transwell migration assays. 36 h after transfection, SCC-25 cells were treated with 10 *μ*M Y27632 for 12 h. Next, cells were seeded in the upper uncoated chamber. DMEM medium (containing 10% FBS) was added to the lower chamber. After 12 h, migratory cells on the lower surface of the chamber were stained and counted under a microscope.

### 2.7. Cell Invasion Assays

Cell migration and invasion assays were performed using transwell chambers (Corning, USA). Transfected SCC-25 cells were seeded in the upper matrigel-coated chamber. DMEM medium (containing 10% FBS) was added to the lower chamber. After 12, 24, and 48 h, cells on the surface of upper chamber were removed using cotton buds. Cells that invaded the lower surface were fixed, stained with crystal violet, and counted under a microscope.

### 2.8. RNA Extraction and RT-qPCR

Total RNA was isolated from tissues or cells by TRIzol reagent (Invitrogen, CA, USA), and the cDNA of HIF-1*α* and ROCK1 were synthesized using a QuantiTect reverse transcription kit (Takara Biotechnology, Tokyo, Japan). GAPDH was selected as endogenous controls. RT-qPCR was performed with KAPA SYBR Fast qPCR Kit (KAPA Biosystems, MA, USA), and the data were normalized relative to GAPDH. The primer sequences were purchased from Invitrogen (Invitrogen, CA, USA), and the relative differences between the assayed groups were calculated with REST MCS software. The primer sequences were as follows: LOC441178:* Forward* GGGTATTTTGTGCTCCCCCA;* Reverse* CAGGCACTGAAGGTTCGGAT; HIF-*α*:* Forward* ATCCATGTGACCATGAGGAAATG;* Reverse* TCGGCTAGTTAGGGTACACTTC; human GAPDH:* Forward* AAGACCTTGGGCTGGGACTG;* Reverse* ACCAAATCCGTTGACTCCGA; ROCK1:* Forward* AACATGCTGCTGGATAAATCTGG;* Reverse* TGTATCACATCGTACCATGCCT;

### 2.9. Western Blot Analysis

Cells were lysed using RIPA lysing buffer (Beyotime, Beijing, China), and protein concentration was measured using a BCA protein assay kit (Beyotime, Beijing, China). Proteins were separated using SDS-PAGE and blotted onto PVDF membranes, which were incubated with primary ROCK-1 antibodies (1:1000, Abcam) for 12 h at 4°C and then with secondary antibodies for 1 h at 37°C. Bands were imaged using the FluorChem E data system (ProteinSimple, CA, USA). The relative density values were determined based on the GAPDH expression level. Each assay was repeated at least three times.

### 2.10. Immunohistochemistry and HE Staining

Immunohistochemistry staining was performed using human OSCC tissue samples and normal tissues. These samples were from high-grade (II-III) OSCC patients. Tissues were first fixed with 4% paraformaldehyde and were then embedded in paraffin and sectioned to 5-*μ*m thickness, and EDTA was used for antigen retrieval. After being mounted on microscope slides, sections were permeabilized with 0.5% Triton X-100 and blocked with rabbit serum. Endogenous peroxidase was quenched by incubating the slides with methanol containing 3% hydrogen peroxide. Next, the sections were incubated with ROCK1 antibody (Abcam) at 4°C overnight. After being rinsed with PBS, the sections were incubated with a horseradish peroxidase-linked antibody. The color reaction was performed using AEC color kit (Sangon Biotech, China). HE staining was performed at the pathology department of Liaocheng People's Hospital.

### 2.11. Statistical Analysis

The results were analyzed using GraphPad Prism 6.01 and the SPSS 12.0 software. Student's* t*-test or a one-way ANOVA was performed to assess the significance of differences. All experimental data were expressed as the means ± SD. A* p* value of < 0.05 indicates a significant difference.

## 3. Results

### 3.1. LOC441178 Was Downregulated in OSCCs Tissues and the Survival Rate of OSCC Patients Was Suppressed

**Figure 1:**
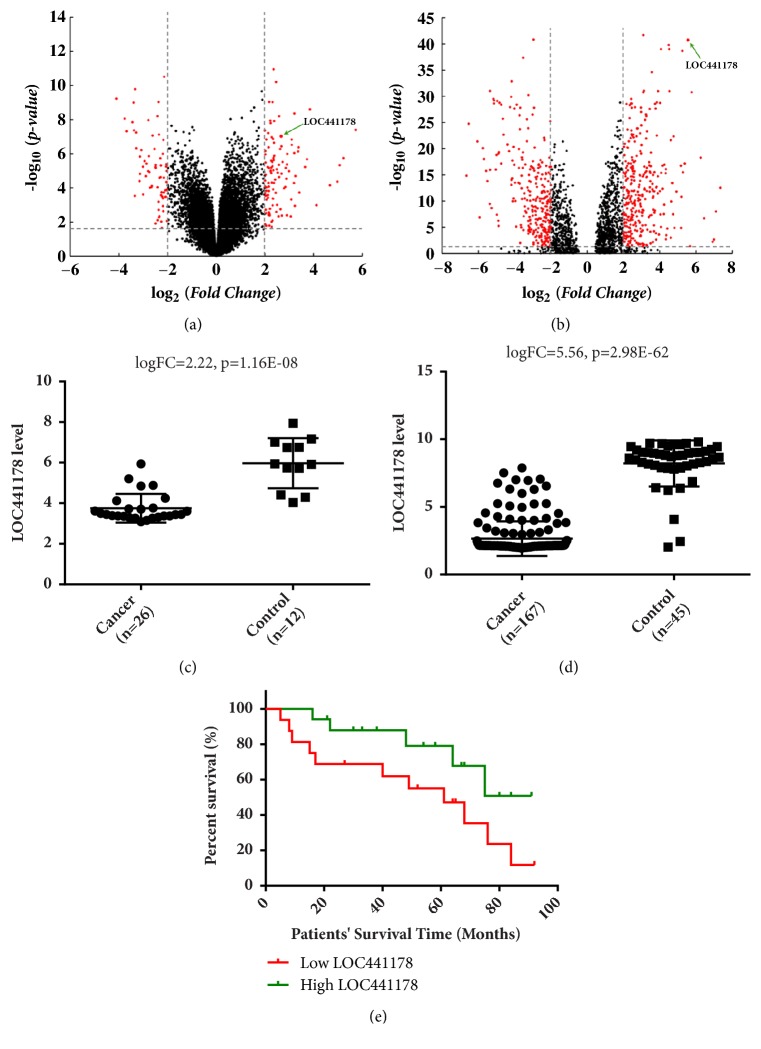
LncRNA array analysis revealed downregulation of LOC441178 in OSCCs. (a) Volcano plots were drawn to show the differentially expressed lncRNAs between OSCCs and control group in GSE9844. Four times fold changes, and a p value of 0.05 was used as division standard. (b) Volcano plots of GSE30784. LOC441178 is one of the most significant lncRNAs with a greater than 14-fold downregulation. (c) The LOC441178 expression levels in OSCC samples were significantly lower than control group in GSE9844. *∗*p < 0.05. (d) The LOC441178 expression levels in GSE30784. (e) The Kaplan-Meier survival curve showed that OSCC patients with low LOC441178 level (red line, n=33) owned a shorter postoperation survival time compared to the high expression subgroup.

Two microarray datasets, GSE30784 and GSE9844, were used to recognize different lncRNAs between OSCC tissues and control groups in expression. In GSE30784, 36 lncRNA have significant differences and 31 of them were downregulated. The expression level of LncRNA LOC441178 in OSCC group was downregulated more than 14 times compared to control group. This result was validated by dataset GSE9844. To make the gene expression differences between OSCC and control group more intuitive, we drew Volcano Plot with criteria of p value < 0.05 (Figures 1(a) and 1(b)). p value has been adjusted by FDR method. LOC441178 expression was significantly lower in the OSCC group than in control group (Figures 1(c) and 1(d)). The results also demonstrated that depressed LOC441178 level reduced the survival rate of OSCC patients, while OSCC patients with high LOC441178 level owned a longer postoperation survival time (Figure 1(e)). These results demonstrated that low LOC441178 level was closely related to poor prognosis.

### 3.2. LOC441178 Knockdown Enhanced the Invasive and Migratory Capacities of SCCs

**Figure 2:**
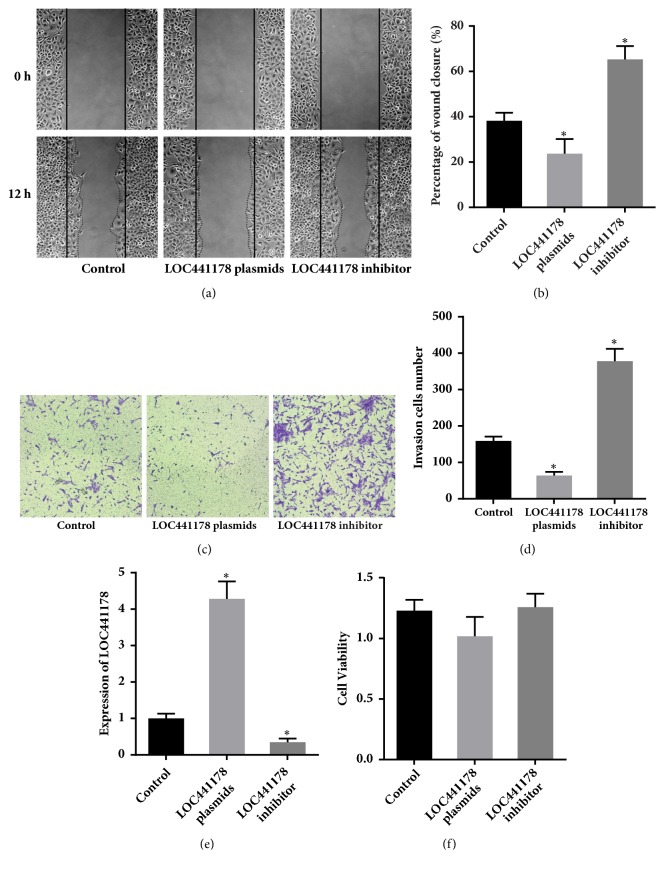
LOC441178 knockdown and overexpression affected the migratory capacities of SCCs. (a and b) Wound-healing assay of transfected SCC-25 cells. A wound was made using a pipette tip, and the gap area size was measured after 12 h. (c) The proinvasive effect of the LOC441178 inhibitor on SCC-25 cells was verified by matrigel invasion assays. The anti-invasive effect of the LOC441178 on SCC-25 cells invasion was also examined. (d) LOC441178 inhibitor significantly enhanced the cell invasion, while LOC441178 overexpression inhibited cell invasion. *∗*p < 0.05. (e) The knockdown and overexpression efficiency of LOC441178 by inhibitor and plasmids in SCC-25 cells were examined by RT-qPCR. *∗*p < 0.05* versus* control. (f) Cell viability assay of SCC-25 cells transfected with the LOC441178 inhibitor or LOC441178 plasmids.

To investigate whether LOC441178 downregulation stimulates SCCs migration, we used a synthetic 2′-O-methyl-modified LOC441178 inhibitor, which is single-stranded antisense oligonucleotides (ASOs) designed to interfere with the 5′-region of LOC441178 by sequestering them via irreversible binding. It contains 19 ribonucleotides and the five ribonucleotides at both ends were 2′-O-methyl-modified. Fully phosphorothioate modification was also adopted to promote the localization of ASOs to the nucleus [15]. RT-qPCR was used to measure the knockdown efficiency of the LOC441178 inhibitor (Figure 2(e)). The results confirmed that the LOC441178 inhibitor had high efficiency and had little impact on the SCCs viability (Figure 2(f)). The wound healing assay revealed that the LOC441178 inhibitor significantly promoted the migration of SCC-25 cells. Hypoxia is a common feature in tumors due to relatively inadequate blood supply and a prognostic factor for poor survival. ROCK1 is a hypoxia-induced kinase, so we examined the expression of HIF-1*α* using RT-PCR (Figure 4(a)). The results showed that the expression of HIF-1*α* was significantly increased after LOC441178 was inhibited.

In order to investigate the proinvasion effects of the LOC441178 inhibitor, matrigel invasion assays were performed to examine SCC-25 cells. Consistent with the wound-healing assay results, similar results were observed. In summary, these results demonstrate that LOC441178 knockdown significantly promotes the invasive and migration capacities of SCCs.

### 3.3. LOC441178 Suppresses the Migration and Invasive Capacities of SCCs by Targeting ROCK1

In order to find out the mechanisms behind the tumor suppressive function of LOC441178, pcDNA3.1 plasmid was used to construct LOC441178 overexpression models. RT-qPCR was performed to verify the overexpression of LOC441178 by transient transfection using pcDNA3.1 plasmid (Figure 2(e)). At the same time, the cell viability was not affected. What is more, a significant change of migration abilities of the transfected SCC-25 cells was observed (Figure 2(f)). The wound healing assay revealed that LOC441178 overexpression significantly inhibited SCCs migration (Figure 2(a)). Matrigel invasion assays were also performed and the same results were observed (Figures 2(c) and 2(d)). Thus, LOC441178 overexpression clearly inhibited the invasive and migratory capacities of SCCs.

Furthermore, we utilized Affymetrix Human Genome U133 Plus 2.0 array, as mentioned previously, to investigate whether LOC441178 functioned through the RhoA/ROCK pathway. The analysis of the results suggested that ROCK1 was probably the target of LOC441178. RT-qPCR was conducted to verify this finding (Figure 4(a)), and we found that LOC441178 knockdown will increase the level of ROCK1. Next, we used the ROCK1 inhibitor Y-27632 to study whether the inhibition of RhoA/ROCK pathway would block the promigration effect of the LOC441178 inhibitor. SCC-25 cells were treated with Y-27632 for 12 h, and subsequent Transwell assay results revealed that ROCK1 inhibition would suppress the promigration effect of LOC441178 inhibitor (Figure 4(b)). In cells that LOC441178 was over expressed, Y-27632 will enhance the antimigration effect slightly (Figure 4(c)).

**Figure 3:**
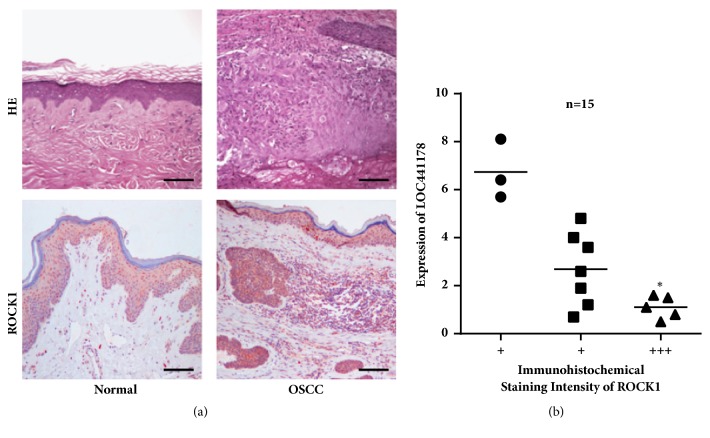
The negative correlation between LOC441178 level and ROCK1 level in OSCC tissues. (a) The immunohistochemical staining of ROCK1 in OSCC tissues from patients. (b) The LOC441178 level is negatively correlated with the ROCK1 level in OSCC tissues. “+” means the intensity of ROCK1 in the immunohistochemical staining. *∗*p < 0.05. Scale bar: 100 *μ*m.

**Figure 4:**
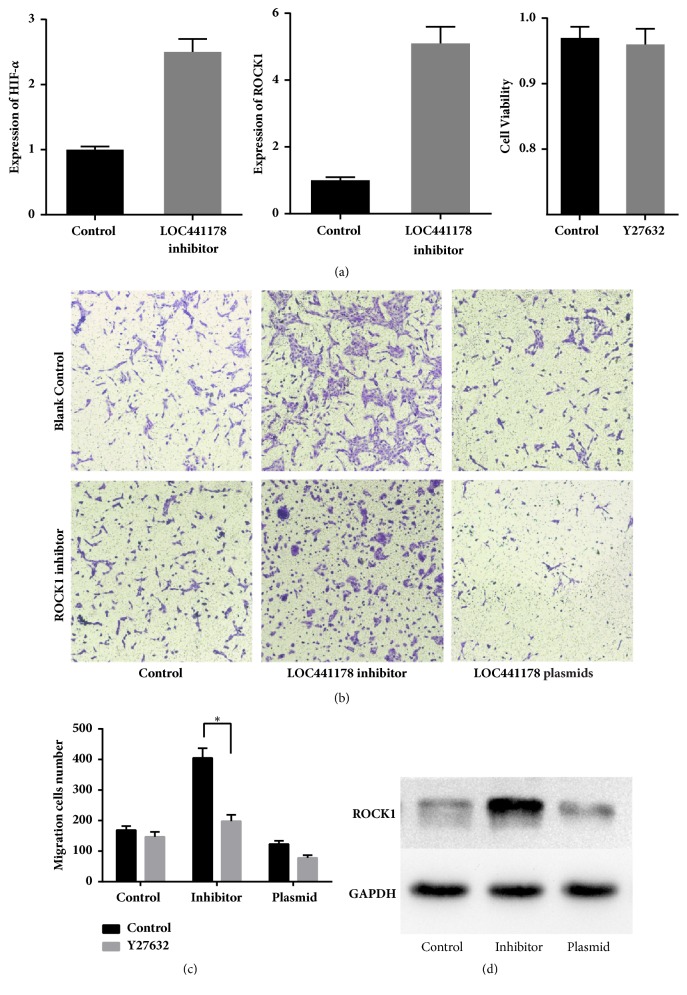
ROCK1 is a target gene of LOC441178. (a) The expression of HIF-1*α* and ROCK1 after LOC441178 knockdown in SCC-25 cells was examined by RT-qPCR. The right panel shows the results of cell viability assay of SCC-25 cells treated with 10 *μ*M Y-27632 for 12 h. *∗*p < 0.05, as determined by Student's* t*-test, for groups* versus* control. The right diagram shows the results of cell viability assay of SCC-25 cells treated with 10 *μ*M Y-27632 for 12 h. (b and c) Effects of ROCK1 inhibitor Y27632 on differently transfected SCC-25 cells were examined by transwell migration assays. The data was shown as mean ± SD. *∗*p < 0.05* versus* control. (d) The ROCK1 levels in transfected SCC-25 cells were examined by western blot. GAPDH was used as control.

To further examine the relationship between LOC441178 expression and ROCK1 level in OSCC, we detected ROCK1 expression in 28 OSCC tissues from patients by western blot. Results demonstrated that LOC441178 expression was negatively correlated with the ROCK1 level in the tumor tissues (Figure 4(d)). The expression of ROCK1 in another 15 OSCC tissue samples from patients was determined by RT-qPCR (Figure 4(a)). In samples that ROCK1 was highly expressed, the expression of LOC441178 was significantly lower (Figures 3(a) and 3(b)). These results demonstrated that the LOC441178 functions by targeting ROCK1 and is likely to be a prognostic biomarker.

## 4. Discussion

In recent years, the functions of lncRNAs in tumor have aroused wide interest, and many researchers have found the potentials of these lncRNAs as therapeutic targets or prognostic indicators [16]. A handful of lncRNAs have been discovered with results showing that they play a role as transcriptional and posttranscriptional regulators, including HOTAIR [17], MALAT1 [9], MEG3 [18], and UCA1 [19]. Here, we found that the expression level of lncRNA LOC441178 was higher in OSCC tissues than corresponding normal tissues using a lncRNA microarray. So LOC441178 was selected as a potential tumor suppressor.

The signaling pathways behind the tumor suppressive role of LOC441178 are poorly studied. We performed functional analyses to prove that LOC441178 antagonizes the invasive and migratory capacities of SCCs, while LOC441178 knockdown significantly enhanced the invasive and migratory capacities of SCCs. Because invasive and migratory capacities are related to fiber formation, which is related to ROCK pathway activation [20], we examined the expression level of this target by RT-qPCR. Next, ROCK1 inhibitor Y27632 was used to test its functional role in this pathway. As the results showed, this inhibitor blocked the promigratory effect of the LOC441178 inhibitor. Subsequently, we examined the ROCK1 protein levels in LOC441178 plasmids transfected or LOC441178 inhibitor transfected SCC-25 cells. The results demonstrated that ROCK1 level was significantly decreased in the former case and elevated in the latter case. All these results supported the conclusion that ROCK1 is the target of LOC441178. We also found that the expression of HIF-1*α* was enhanced after LOC441178 was inhibited,

In conclusion, our results showed the anti-invasive and antimigratory effects of LOC441178 in OSCC, which can be a new ROCK1 inhibitory lncRNA, and suggest that it may be a prognostic biomarker. All these findings may benefit the development of lncRNA-related diagnosis and treatment in OSCC.

## Data Availability

The data used to support the findings of this study are included within the article. The gene expression data can be accessed on Gene Expression Omnibus (GEO).
